# Mechanically processed, vacuum- and etch-free fabrication of metal-wire-embedded microtrenches interconnected by semiconductor nanowires for flexible bending-sensitive optoelectronic sensors

**DOI:** 10.1515/nanoph-2023-0667

**Published:** 2024-01-11

**Authors:** Taeyun Kim, Minwook Kim, Jinkyu Han, Hocheol Jeong, Seungmin Lee, Jaeil Kim, Daeho Lee, Hoon Eui Jeong, Jong G. Ok

**Affiliations:** Department of Mechanical and Automotive Engineering, Seoul National University of Science and Technology, 232 Gongneung-ro, Nowon-gu, Seoul 01811, Republic of Korea; Department of Mechanical Engineering, Gachon University, 1342 Seongnamdaero, Sujeong-gu, Seongnam, Gyeonggi 13120, Republic of Korea; Department of Mechanical Engineering, Ulsan National Institute of Science and Technology, Ulsan 44919, Republic of Korea

**Keywords:** mechanical patterning, microtrench, metal wire embedding, semiconductor, zinc oxide nanowire, optoelectronic transducer

## Abstract

We demonstrate the facile fabrication of metal-wire-embedded microtrenches interconnected with semiconducting ZnO nanowires (ZNWs) through the continuous mechanical machining of micrograting trenches, the mechanical embedding of solution-processable metal wires therein, and the metal-mediated hydrothermal growth of ZNWs selectively thereto. The entire process can be performed at room or a very low temperature without resorting to vacuum, lithography, and etching steps, thereby enabling the use of flexible polymer substrates of scalable sizes. We optimize the fabrication procedure and resulting structural characteristics of this nanowire-interconnected flexible trench-embedded electrode (NIFTEE) architecture. Specifically, we carefully sequence the coating, baking, and doctor-blading of an ionic metal solution for the embedding of clean metal wires, and control the temperature and time of the hydrothermal ZNW growth process for faithful interconnections of such trench-embedded metal wires *via* high-density ZNWs. The NIFTEE structure can function as a bending-sensitive optoelectronic sensor, as the number of ZNWs interconnecting the neighboring metal wires changes upon mechanical bending. It may benefit further potential applications in diverse fields such as wearable technology, structural health monitoring, and soft robotics, where bending-sensitive devices are in high demand.

## 1 Introduction

In recent years, there has been growing interest in the development of optoelectronic transducers utilizing micro- and nano-scale materials and structures to serve various functions, including sensing, communication, and energy conversion, in compact, lightweight, and flexible forms [1]–[7]. In particular, optoelectronic micro- and nano-architectures, which consist of micropatterned electrodes interconnected by the semiconductor nanowires (NWs) such as ZnO NWs (ZNWs), have attracted significant attention due to their high sensitivity to incident light with specific intensity and wavelength [6]–[17]. However, the conventional methods for fabricating micropatterned electrodes mostly involve complex and time-consuming processes, such as vacuum deposition, optical lithography, and etching, which often limit their scalability and widespread use [7], [17–24]. Additionally, ZNW growth that typically relies on high-temperature seed sintering or chemical vapor deposition may block its practical application to flexible substrates [25]–[30].

One smart approach to overcoming the aforementioned challenges is to create a micropatterned electrode *via* a mechanical process, for instance by machining microtrenches, mechanically filling the metal therein, and then growing ZNWs selectively on the embedded metal wires *via* a sintered-seed-free hydrothermal process. Working on this novel strategy, we develop a vacuum- and etch-free method for the fabrication of metal-wire-embedded microtrenches interconnected by ZNWs. Our method involves the continuous mechanical inscribing of linear microtrench patterns (microgratings) on a substrate, followed by the doctor-blade-assisted embedding of solution-processable metal wires within those trenches [31]. We then undertake the low-temperature metal-mediated hydrothermal growth of ZNWs selectively onto the metal wires, finalizing the ZNW-interconnected micrograting electrode structure. The entire process can be carried out at a low temperature without resorting to vacuum, lithography, and/or etching steps, thereby enabling the use of flexible polymer substrates of scalable sizes. The resulting flexible device can function as a bending-sensitive optoelectronic sensor with high sensitivity, as the number of ZNWs interconnecting the trench-embedded micrograting electrodes changes upon mechanical bending [29], [32], [33].

## 2 Results and discussion

**Figure 1:**
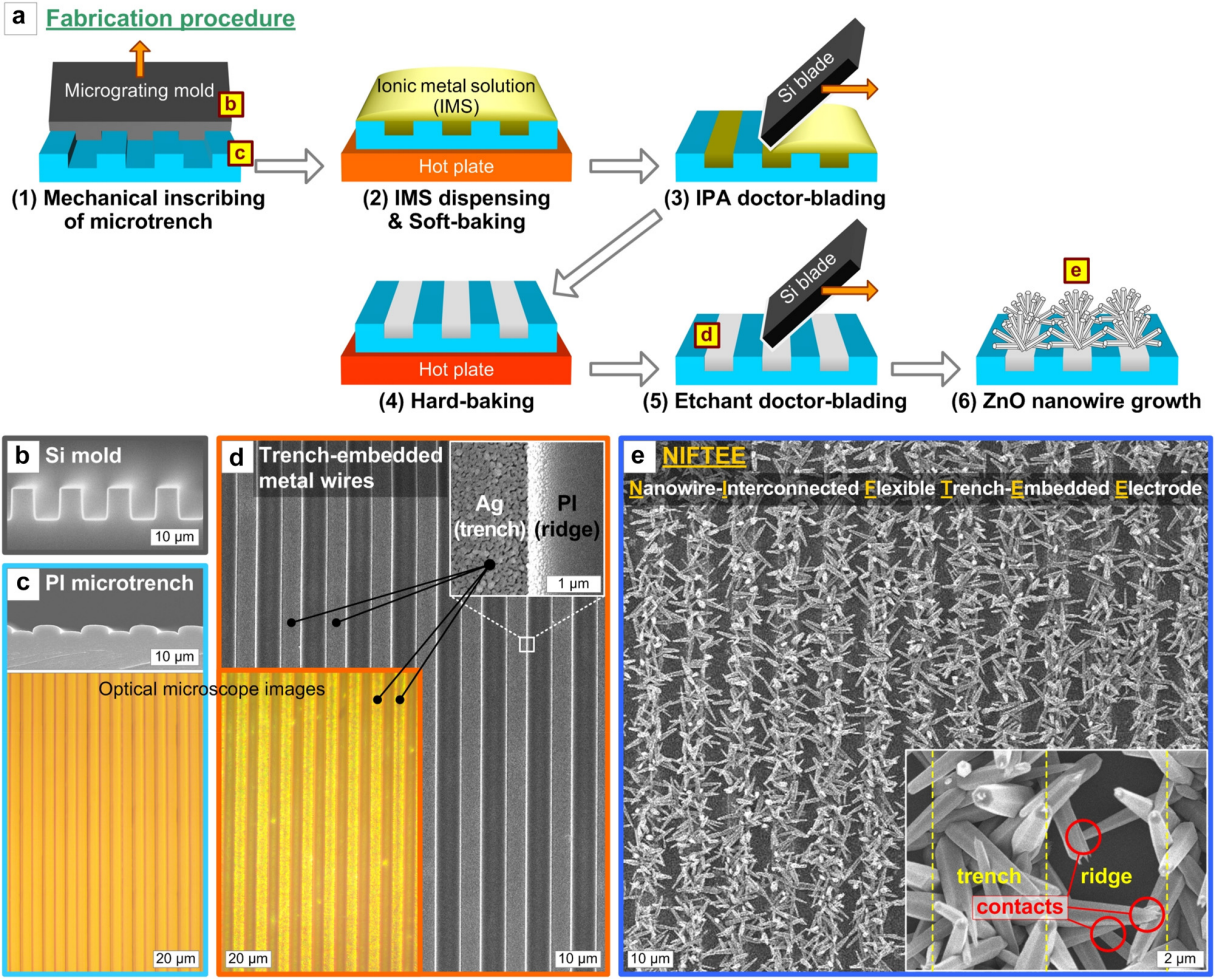
Fabrication of the nanowire-interconnected flexible trench-embedded electrode (NIFTEE) structure. (a) Schematic illustration of the NIFTEE fabrication procedure, which consists of the mechanical inscribing of a microtrench pattern structure, the solution-based mechanical embedding of metal wires therein, and the selective growth of ZNWs thereto. Representative scanning electron microscope (SEM) and optical microscope images of the fabrication results of each step: (b) Si tool used for mechanical machining of microtrenches, (c) PI microtrench pattern, (d) trench-embedded metal (Ag) wires, and (e) ZNWs grown on and then interconnecting the metal wires. The upper-right inset of (d) shows an enlarged SEM view of the boundary region between the trench-embedded Ag wire and the PI ridge. The lower-right inset image of (e) is a high-resolution SEM view showing that the neighboring metal wires are interconnected *via* ZNW-ZNW contacts.

Figure 1a depicts the overall procedure for the fabrication of the nanowire-interconnected flexible trench-embedded electrode, termed NIFTEE, showing the continuous mechanical inscribing of microtrench patterns on a flexible substrate, the solution-processed embedding of metal wires therein, and the low-temperature metal-mediated hydrothermal growth of ZNWs thereto. First, for the mechanical machining of microtrenches, a sliced edge of a 10-µm-period Si micrograting tool, with the grating ridge’s height and duty (*i.e.*, width ratio of ridge to inter-ridge opening) of 5 µm and 1:1, respectively (Figure 1b), makes conformal contact with and slides over a flexible substrate, in this case polyimide (PI) film, at a controlled contact angle (∼35°) and force (∼4 N), tool temperature (∼230 °C), and feed rate (1 mm/s). This results in the etch-free fabrication of flexible ∼5-µm-wide microtrench patterns (typically with a ∼1 cm-long and ∼2 cm-wide area) with a period and depth of 10 µm and ∼1.5 µm, respectively (Figure 1c), in a continuous and scalable manner. The period, depth, and profile of the microtrench can be modulated by varying the tool’s geometry and temperature, contacting angle and force, and inscribing speed, as previously studied in detail [34], [35].

The procedure for embedding metal wires in the microtrenches consists of the following steps: (1) dispensing an ionic metal solution (IMS), a mixture of ionic metal species (Ag-amine compounds in this work) and solvent (isopropyl alcohol (IPA) in this work), (2) soft-baking (90 °C, 1 min) to semi-solidify the dispensed IMS by reducing the solvent, (3) scraping off the IMS by the doctor-blade method using a Si blade wrapped with an IPA-soaked fab wipe (simply referred to as ‘IPA doctor-blading’ hereafter), (4) hard-baking (150 °C, 5 min) to reduce the trench-embedded IMS to the metal (Ag) wire, and (5) another doctor-blade step using an Ag-etchant-soaked fab-wipe-wrapped Si blade (‘etchant doctor-blading’ hereafter). Experimental details of each step are fully described in the Supplementary Material. This mechanical metal-wire-embedding process, which may be central to the NIFTEE fabrication, can be optimized by carefully sequencing the IMS baking and doctor-blading processes.

The IMS turns into a gel-like phase upon soft-baking, which helps with the facile mechanical scraping of the surficial IMS off the surface by IPA doctor-blading while leaving the trench-embedded IMS. Without soft-baking, the IMS remains as a liquid and can nearly be simply swept away during the IPA doctor-blading step due to its overly rapid absorption into the fab wipe. Once the IMS is fully reduced to the metal by hard-baking, IPA doctor-blading may not easily peel off the surficial part owing to the increased mechanical stiffness. This is why IPA doctor-blading should be performed between the soft-baking and hard-baking processes, a strategy similar to that in our previous work where we used an Ag-nanoparticle-dispersed colloidal solution instead of IMS [31]. The last step, etchant doctor-blading, is essential for cleaning the residual Ag species from the top surficial region (*i.e.*, ridge) and polishing the trench-embedded Ag wires (Figure 1d). Although the appearance of the surface is seemingly identical before and after etchant doctor-blading, this is crucial for growing ZNWs selectively on the trench-embedded metal wire electrodes (Figure 1e), as will be comparatively demonstrated in the following paragraphs.

Finally to complete the NIFTEE structure, flexible trench-embedded metal microgratings, not connected to one another up to this point, can be interconnected by growing conformally oriented ZNWs thereto. Growing ZNWs selectively on the metal wires is enabled by a low-temperature (∼90 °C) hydrothermal process *via* a metal-mediated growth mechanism [26], [36], [37]; the metal (Ag) surface provides a favorable condition for the nucleation and continued growth of ZnO crystals in an aqueous precursor bath containing zinc nitrate (Zn(NO_3_)_2_) and HMTA (hexamethylenetetramine; C_6_H_12_N_4_). More specifically, in the precursor solution that is generally alkaline because of HMTA, Zn species tend to form negatively charged complexes (*e.g.*, Zn(OH)_3_^−^ and Zn(OH)_4_^2−^); the Ag surface with the native oxide layer is positively ionized in the solution and thus attracts the negatively charged Zn complexes, thereby promoting the nucleation of ZnO; the great lattice match between the oxidized Ag surface and ZnO facilitates continued growth of ZnO crystals towards conformal ZNWs [38]. This obviates a high-temperature (∼350 °C)-sintered ZnO seed and thus allows the use of flexible substrates. The full experimental method can be found in the literature [36, 37].

**Figure 2:**
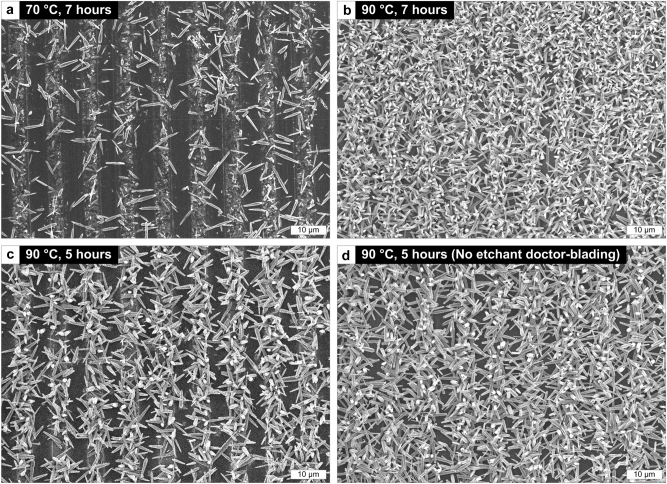
SEM images of parametrically controlled NIFTEE structures fabricated by growing ZNWs at various temperatures and times *via* a metal-mediated hydrothermal process: (a) 70 °C and 7 h, (b) 90 °C and 7 h, and (c) and (d) 90 °C and 5 h. The etchant doctor-blading step was skipped on purpose for (d).

The density and length of the ZNWs can be tailored specifically to interconnect the metal wires suitably by controlling the temperature and growth time of the hydrothermal process. Several cases are shown in Figure 2; the temperature and growth time are varied within the corresponding ranges of 70–90 °C and 5–7 h, respectively. Under the condition of 70 °C and 7 h, the density of the ZNWs is overall not very high due to the less active hydrothermal reaction at the lower temperature in this case (Figure 2a). This can cause unsatisfactory interconnections between the metal wires. High-density conformal ZNWs can be readily grown at a temperature higher than 90 °C, though too long a growth time (*e.g.*, 7 h) causes overly dense ZNWs that undesirably cover nearly the entire surface (Figure 2b). Such an outcome can degrade the function of the metal wires underneath the ZNWs as well as the sensitivity of the NIFTEE-based sensor device. By adjusting the growth time to 5 h in our parametric space, we find that the trench-embedded metal wires are interconnected by the ZNWs selectively grown thereto to an adequate density and length (Figure 2c). The etchant doctor-blading method discussed above is useful particularly in this case for securing the cleanliness of the inter-trench ridges with few ZNWs, which would otherwise be prone to ‘weedy’ ZNWs due to the residual Ag species. Figure 2d reveals a counterexample in which etchant doctor-blading was not done.

**Figure 3:**
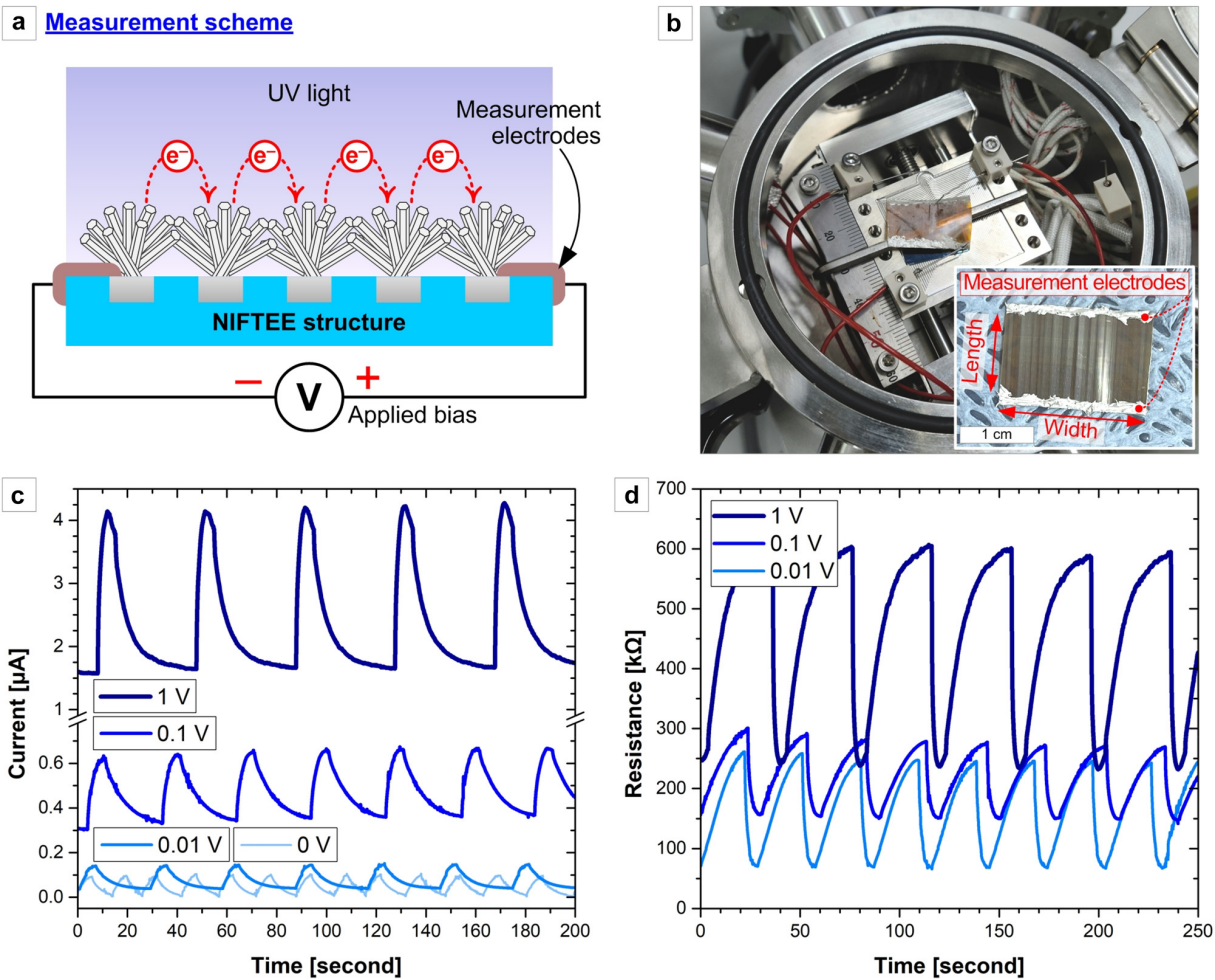
Characterization of the NIFTEE architecture as a reliable flexible UV light sensor: (a) Schematic drawing of measurement and (b) photograph of actual measurement. The lower-right inset of (b) shows a 1 × 2 cm-sized NIFTEE sample with Ag-pasted measurement electrodes. (c) Photocurrents and (d) resistance values (*versus* time) measured at various applied biases upon pulsed UV light illumination onto the NIFTEE device. The UV light intensity was set to 700 mW/cm^2^ for all measurements.

The NIFTEE architecture with ZNWs (grown at 90 °C for 5 h, unless otherwise noted) can be used as a UV light sensor, where its unique vacuum- and etch-free mechanical processability and low-temperature fabricability collectively enable cost-effective and scalable device fabrication. Figure 3a and b show the measurement scheme and the actual measurement setup, respectively, for a NIFTEE device with its length and width of ∼1 cm and ∼2 cm, respectively (see the inset of Figure 3b). After measurement electrodes are fabricated at both ends of the NIFTEE device using Ag paste, electric bias voltage at various levels (0, 0.01, 0.1, and 1 V) can be applied across the micrograting axis so that the electrons can ‘hop’ (see Figure 3a) across the metal wires through the ZNW-ZNW contact points. Upon illumination with UV light, the extra free electrons photogenerated in the n-type semiconductor ZNWs can be collected as photocurrent through the measurement electrodes.

Figure 3c and d show the currents and device resistance values, respectively, measured at various applied bias voltages upon illumination with pulsed UV light (365 nm peak wavelength, ∼700 mW/cm^2^). Notably, a measurable current can be collected at zero bias, indicating that the free electrons could be readily photogenerated in ZnO. It is clearly shown that the UV-light-induced photocurrent increases and the device resistance decreases as the bias voltage increases. Although the cross-sectional area of the NIFTEE device may not be conventionally defined for calculation of quantitative current density values, we assume the maximum cross-area by taking its width and height as 2 cm (device width) and 6.5 µm (sum of the average length of ZNWs (∼5 µm) and the depth of trench-embedded Ag wire (∼1.5 µm)), respectively. Even without any external bias, we obtain the maximum current density (*J*_max_) of 9.23 × 10^−2^ mA/cm^2^, the minimum current density (*J*_min_) of 9.08 × 10^−4^ mA/cm^2^, and the average current density (*J*_*avg*_) of 3.75 × 10^−2^ mA/cm^2^. At 1 V bias, the NIFTEE device exhibits *J*_max_ of 3.37 mA/cm^2^, *J*_min_ of 1.20 mA/cm^2^, and *J*_*aver*_ of 1.78 mA/cm^2^. The ratio of the change in the resistance (*ΔR*) due to the incident UV light to the original value (*R*_*0*_) with no UV light, *ΔR*/*R*_*0*_, is in range of 0.5–0.75, which confirms that the NIFTEE structure functions as a sensitive UV sensor.

**Figure 4:**
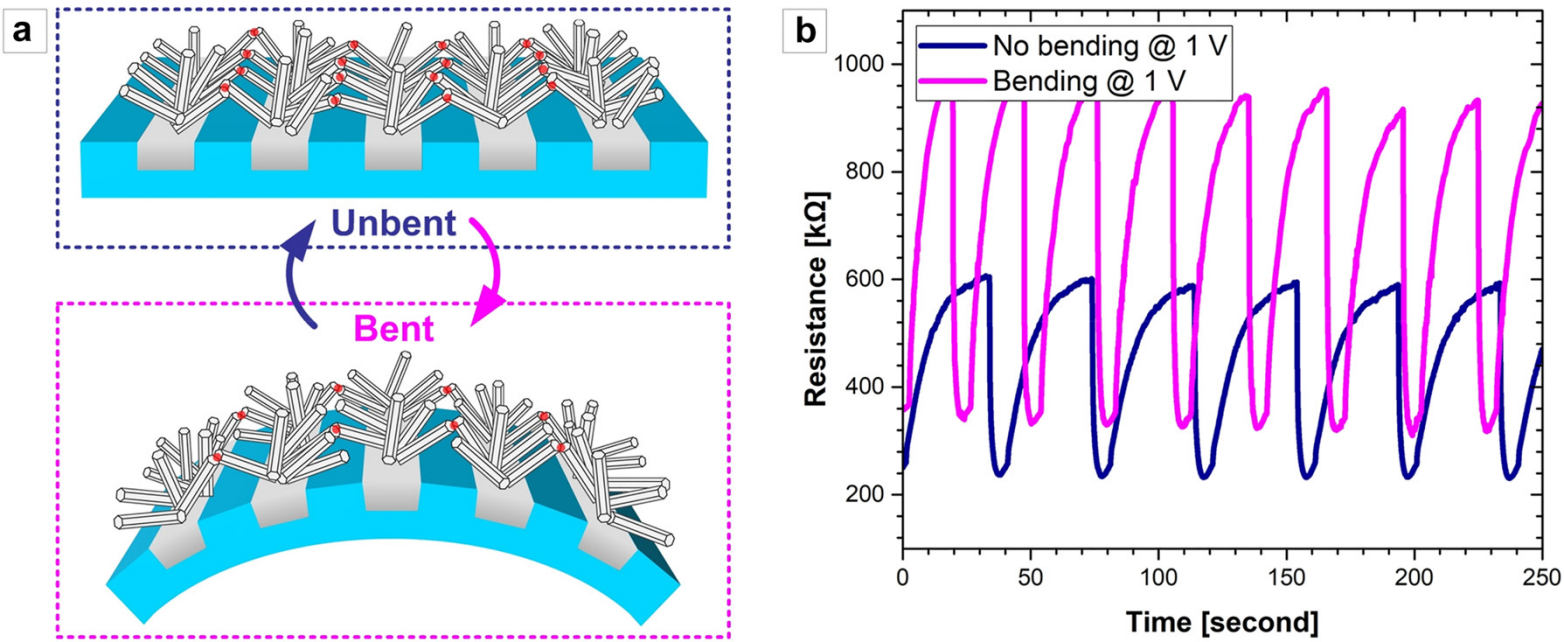
Characterization of the NIFTEE architecture as a bending-sensitive UV light sensor: (a) schematics of normal (unbent; left) and bent (right) NIFTEE devices, comparatively depicting the difference in the number of ZNWs interconnecting the neighboring metal wires. The ZNW-ZNW contact points are illustrated with red dots. (b) Resistance values (*versus* time) measured at 1 V bias for unbent and bent NIFTEE devices. The bending radius was ∼5 mm. The UV light intensity was set to 700 mW/cm^2^ for all measurements.

When this flexible structure is bent, the number of ZNW-ZNW contacts interconnecting the metal wires to one another can change, which consequently changes the overall device resistance and thus modulates the sensitivity for incident UV light. Figure 4a schematically illustrates a convexly bent state (with the bending radius of ∼5 mm) of a NIFTEE device compared to a normally unbent sample. Figure 4b comparatively shows the changes in resistance for both unbent and bent operation at a bias of 1 V. Because some ZNWs lose their contacts from others as the device is bent, the device resistance increases. However, it can also be found that the device sensitivity (*ΔR*/*R*_*0*_) improves because the contribution of UV-light-induced photogenerated electrons to the overall device resistance becomes more pronounced. This suggests that the NIFTEE architecture can function not only as a flexible bending-sensitive optoelectronic sensor but that it can also be utilized to detect structural deformations.

We currently hit upon another interesting idea that the flexible NIFTEE device, once created on a large area, can be cut simply with scissors to tailor the length and total number of ZNW-interconnected metal wires. This can be realized exclusively because the NIFTEE structure does not involve any specific electrode patterns, such as an interdigitated electrode; it is merely a simple array of paralleled metal wires which can still work after being cut to any length and width. This will allow the practical mass-manufacturing of multiple devices with controlled performance and sensitivity levels without repeated design and fabrication steps. This study is currently in progress.

## 3 Conclusions

In summary, we have developed the NIFTEE architecture: ZNW-interconnected trench-embedded metal wires, through a vacuum- and etch-free mechanical process consisting of the dynamic inscribing of a linear microtrench array, tactfully sequenced IMS baking and doctor-blading steps, and metal-mediated hydrothermal ZNW growth. We demonstrated that the NIFTEE structure can be fabricated on a flexible substrate at a low temperature and that it can perform as a bending-sensitive optoelectronic sensor by regulating the number of ZNWs that maintain contact between adjacent metal wires upon mechanical bending. Our approach offers a “nifty” method for the fabrication of flexible transducers with an excellent scalability for mass production. Many potential applications can benefit from our approach, including but not limited to wearable devices, environmental sensors, and structural health monitors.

## Supplementary Material

Supplementary Material Details

## References

[1] SergentTakiguchi S. M., Tsuchizawa T., Taniyama H., Notomi M. (2019). ZnO-Nanowire-Induced nanocavities in photonic crystal disks. *ACS Photonics*.

[2] Portone A. (2020). Conformable nanowire-in-nanofiber hybrids for low-threshold optical gain in the ultraviolet. *ACS Nano*.

[3] Zhang Y. Z. (2020). Enhanced photovoltaic performances of La-doped bismuth ferrite/zinc oxide heterojunction by coupling piezo-phototronic effect and ferroelectricity. *ACS Nano*.

[4] Zhu Y., Raj V., Li Z. Y., Tan H. H., Jagadish C., Fu L. (2021). Self-powered InP nanowire photodetector for single-photon level detection at room temperature. *Adv. Mater.*.

[5] Dalapati G. K. (2021). Tin oxide for optoelectronic, photovoltaic and energy storage devices: a review. *J. Mater. Chem. A*.

[6] Hu J. (2023). Research advances in ZnO nanomaterials-based UV photode tectors: a review. *Nanotechnology*.

[7] Rekha S. M., Neelamana H. V., Bhat S. V. (2023). Recent advances in solution-processed zinc oxide thin films for ultraviolet photodetectors. *ACS Appl. Electron. Mater.*.

[8] Alenezi M. R., Henley S. J., Silva S. R. P. (2015). On-chip fabrication of high performance nanostructured ZnO UV detectors. *Sci. Rep*..

[9] Yan C. Y., Singh N., Lee P. S. (2010). Wide-bandgap Zn2GeO4 nanowire networks as efficient ultraviolet photodetectors with fast response and recovery time. *Appl. Phys. Lett.*.

[10] Erfan M., Gnambodoe-Capochichi M., Leprince-Wang Y., Marty F., Sabry Y. M., Bourouina T. (2019). Nanowire length, density, and crystalline quality retrieved from a single optical spectrum. *Nano Lett*..

[11] Chen T., Gao X., Zhang J. Y., Xu J. L., Wang S. D. (2020). Ultrasensitive ZnO nanowire photodetectors with a polymer electret interlayer for minimizing dark current. *Adv. Opt. Mater.*.

[12] Li H. H. (2020). Controllable heterogeneous nucleation for patterning high-quality vertical and horizontal ZnO microstructures toward photodetectors. *Small*.

[13] Qiao S., Sun H. J., Liu J. H., Fu G. S., Wang S. F. (2022). The nanowire length dependence of the photoresponse and Pyro-phototronic response in the ZnO-based heterojunctions. *Nano Energy*.

[14] You D. T. (2019). Single-crystal ZnO/AlN core/shell nanowires for ultraviolet emission and dual-color ultraviolet photodetection. *Adv. Opt. Mater.*.

[15] Zhang L. (2022). Large-area flexible and transparent UV photodetector based on cross-linked Ag NW@ZnO NRs with high performance. *J. Mater. Sci. Technol.*.

[16] Ma S. H., Dahiya A. S., Christou A., Dahiya R. (2022). All-printed ZnO nanowire based high performance photodetectors. *2022 IEEE International Conference on Flexible and Printable Sensors and Systems (FLEPS)*.

[17] Chen P., Hang T. (2023). Simple template-mediated fabrication of ZnO nanotube arrays and their application in flexible ultraviolet photodetectors. *ACS Appl. Nano Mater.*.

[18] Zhao X. X. (2020). Synthesis of monodispersedly sized ZnO nanowires from randomly sized seeds. *Nano Lett*..

[19] Alenezi M. R., Almeshal A. M., Alkhaledi A. (2022). Hierarchical zinc oxide nanobrushes ultraviolet photodetector. *Micro Nano Lett.*.

[20] Le A. T., Ahmadipour M., Pung S. Y. (2020). A review on ZnO-based piezoelectric nanogenerators: synthesis, characterization techniques, performance enhancement and applications. *J. Alloys Compd.*.

[21] Zhang B., He J. K., Li J. X., Wang L., Li D. C. (2019). Microscale electrohydrodynamic printing of in situ reactive features for patterned ZnO nanorods. *Nanotechnology*.

[22] Kim M. (2022). Solution-processable, Ag-sandwiched nanotube-coated, durable (SAND) architecture realizing anti-breaking cyclic heating on arbitrary substrates. *Int. J. Precis. Eng. Manuf. Green Technol.*.

[23] Kim M. (2022). Facile fabrication of stretchable photonic Ag nanostructures by soft-contact patterning of ionic Ag solution coatings. *Nanophotonics*.

[24] An Y., Li S.-X., Feng J.-C., Xia H. (2023). Highly responsive, polarization-sensitive, flexible, and stable photodetectors based on highly aligned CsCu_2_I_3_ nanowires. *Adv. Opt. Mater.*.

[25] Narzary R., Chetia R., Sahu P. P. (2023). A low-temperature efficient approach for the fabrication of ZnO-rGO heterostructures for applications in optoelectronic applications. *IEEE Access*.

[26] Oh D. K., Choi H., Shin H., Kim K., Kim M., Ok J. G. (2021). Tailoring zinc oxide nanowire architectures collectively by catalytic vapor-liquid-solid growth, catalyst-free vapor-solid growth, and low-temperature hydrothermal growth. *Ceram. Int.*.

[27] Yang Z. (2015). Developing seedless growth of ZnO micro/nanowire arrays towards ZnO/FeS2/CuI P-I-N photodiode application. *Sci. Rep*..

[28] Alenezi M. R., Almeshal A. M. (2021). Bridging nanowires for enhanced gas sensing properties. *Crystals*.

[29] Kwon D. K., Porte Y., Ko K. Y., Kim H., Myoung J. M. (2018). High-performance flexible ZnO nanorod UV/gas dual sensors using Ag nanoparticle templates. *ACS Appl. Mater. Interfaces*.

[30] Lin C. Y., Li Q. Y., Guang H. Z., An M. Z., Zn E. (2022). A promising alternative to ZnO seed layer for hydrothermal growth of ZnO nanowire array. *Mater. Lett.*.

[31] Lee W. (2021). Solution-processable electrode-material embedding in dynamically inscribed nanopatterns (SPEEDIN) for continuous fabrication of durable flexible devices. *Microsyst. Nanoeng.*.

[32] Bai S., Wu W. W., Qin Y., Cui N. Y., Bayerl D. J., Wang X. D. (2011). High-performance integrated ZnO nanowire UV sensors on rigid and flexible substrates. *Adv. Funct. Mater.*.

[33] Kwon D. K., Lee S. J., Myoung J. M. (2016). High-performance flexible ZnO nanorod UV photodetectors with a network-structured Cu nanowire electrode. *Nanoscale*.

[34] Oh D. K. (2019). Tailored nanopatterning by controlled continuous nanoinscribing with tunable shape, depth, and dimension. *ACS Nano*.

[35] Oh D. K. (2022). Burr- and etch-free direct machining of shape-controlled micro- and nanopatterns on polyimide films by continuous nanoinscribing for durable flexible devices. *Microelectron. Eng.*.

[36] Yoo K. (2020). Low-temperature large-area fabrication of ZnO nanowires on flexible plastic substrates by solution-processible metal-seeded hydrothermal growth. *Nano Convergence*.

[37] Choi H. (2022). Solution-processable Ag-mediated ZnO nanowires for scalable low-temperature fabrication of flexible devices. *ACS Appl. Electron. Mater.*.

[38] Kim B., Kwon J. (2014). Metal catalyst for low-temperature growth of controlled zinc oxide nanowires on arbitrary substrates. *Sci. Rep*..

