# Supplementary material for: Mechanically processed, vacuum- and etch-free fabrication of metal-wire-embedded microtrenches interconnected by semiconductor nanowires for flexible bending-sensitive optoelectronic sensors
Source: Nanophotonics. 2024 Jan 11;13(7):1141–8. doi: 10.1515/nanoph-2023-0667 (PMC11501607; doi:10.1515/nanoph-2023-0667)
Supplement: Supplementary file 1 — Supplementary Material Details [file j_nanoph-2023-0667_suppl_001.docx]

Supplementary Material

**Mechanically processed, vacuum- and etch-free fabrication of metal-wire-embedded microtrenches interconnected by semiconductor nanowires for flexible bending-sensitive optoelectronic sensors**

Taeyun Kim,^1,†^ Minwook Kim,^1,†^ Jinkyu Han,^1^ Hochul Jung,^1^ Seungmin Lee,^1^ Jaeil Kim,^3^ Daeho Lee,^2^ Hoon Eui Jeong,^3,^* and Jong G. Ok^1,^*

^1^ Department of Mechanical and Automotive Engineering, Seoul National University of Science and Technology, 232 Gongneung-ro, Nowon-gu, Seoul 01811, Republic of Korea

^2^ Department of Mechanical Engineering, Gachon University, 1342 Seongnamdaero, Sujeong-gu, Seongnam, Gyeonggi 13120, Republic of Korea

^3^ Department of Mechanical Engineering, Ulsan National Institute of Science and Technology, Ulsan 44919, Republic of Korea

^†^ These authors contributed equally to this work.

*Corresponding authors:

Dr. Jong G. Ok, jgok@seoultech.ac.kr, Tel. +82-2-970-9012

Dr. Hoon E. Jeong, hoonejeong@unist.ac.kr, Tel. +82-52-217-2339

*Experimental procedure for embedding metal wires in the microtrenches*

The ionic metal solution (IMS) used in this study was prepared by diluting a TEC-CO-11 ink (Ag carbonates and carbamates mixed in isopropyl alcohol (IPA); InkTec Co., Ltd.) in more IPA at a volume ratio of 3:2 (corresponding to a volumetric concentration of 60%, unless otherwise specified). A sufficient amount of the IMS was dropped onto the microtrench-patterned surface and was spin-coated (300 rpm, 30 seconds) to ensure uniform spreading. The sample was then soft-baked at 90 °C for one minute on a hot plate. The doctor-blading process was applied to the sample surface using a Si blade wrapped with an IPA-soaked fab wipe piece (IPA doctor-blading) at a contact angle between the blade and the substrate surface of ~35°. The typical doctor-blading speed and the normal force applied to the blade were ~1 mm/s and ~900 gf, respectively. The doctor-blading stroke, typically made in the direction across the micrograting axis, was repeated four times; each time the stroke direction was reversed so that the surface could be doctor-bladed ‘back and forth’, which facilitated more uniform IMS embedding in the trenches. The sample was then hard-baked at 150 °C for five minutes on a hot plate. Finally, the sample surface was doctor-bladed using a Si blade wrapped with an Ag etchant (Type A, Transene)-soaked fab wipe piece (etchant doctor-blading) under conditions identical to those of the IPA doctor-blading method. The sample was gently rinsed with deionized water and dried by gentle nitrogen blowing.
